# CAPTURING REAL-WORLD LOSSES OF BALANCE AND RECOVERY RESPONSES IN OLDER ADULTS AT RISK FOR FALLS

**DOI:** 10.1093/geroni/igz038.030

**Published:** 2019-11-08

**Authors:** Neil Alexander, Shirley Handelzalts-Pereg, Linda Nyquist, Debbie Strasburg, Nicholas Mastruserio, Lauro Ojeda

**Affiliations:** 1 University of Michigan, Ann Arbor, Michigan, United States; 2 Ben gurion University, Israel, Ann Arbor, Michigan, United States

## Abstract

Losses of balance (LOBs) such as trips can lead to falls in older adults; what actually happens during real-world LOBs is unclear. With 4 wearable inertial measurement units (IMUs), we recorded feet, trunk and wrist movements over 2 weeks. Using a wrist voice recorder to report the LOBs, we applied our IMU processing algorithms and reconstructed the full body LOB and recovery motions. We recruited 7 at-risk older adults (M=76 yrs) who reported 114 LOBs of which we reconstructed over 90%. Using a rating system, 52% of the LOBs involved a significant trip, stumble, recovery step, and/or large trunk motion. 25% involved double or stutter steps and smaller trunk motions. The other 23% had less striking associated motions. These data suggest that most, but not all, self-reported real world LOBs involve substantial postural destabilization and near falls. Analyses of the voice-recorded context under which the LOBs occurred are ongoing.

